# Supplementary material for: Impedimetric DNA Sensors for Epirubicin Detection Based on Polythionine Films Electropolymerized from Deep Eutectic Solvent
Source: Sensors (Basel). 2023 Oct 4;23(19):8242. doi: 10.3390/s23198242 (PMC10575168; doi:10.3390/s23198242)
Supplement: Supplementary file 1 [file sensors-23-08242-s001.zip › sensors-2604504-supplementary.pdf]

Electronic Supporting Information

to the article of A.I. Goida, A.M. Rogov, Y.I. Kuzin, A.V. Porfireva, G.A. Evtugyn "Impedimetric DNA-Sensor for Epirubicin Detection Based on Polythionine Films Electropolymerized from Deep Eutectic Solvent"

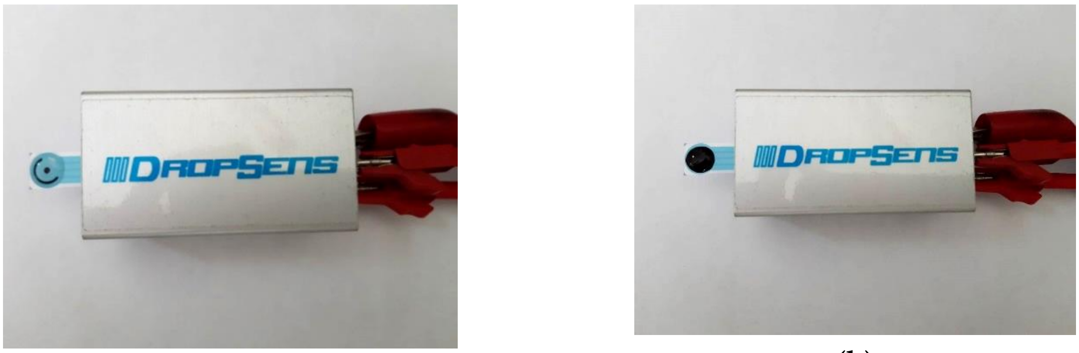

(a) (b)

Figure S1. Electrochemical cell design. (a) NADES is drop-casted on the electrode; (b) NADES with thionine is drop-casted on the electrode

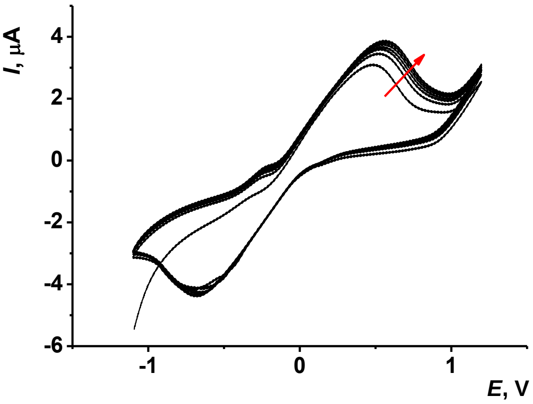

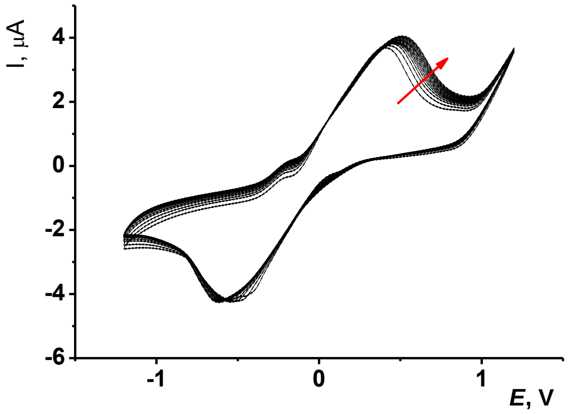

(a) (b)

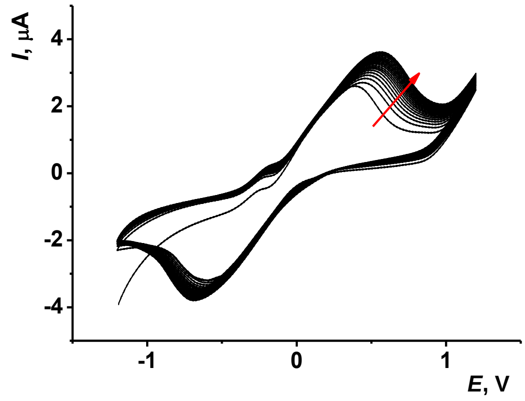

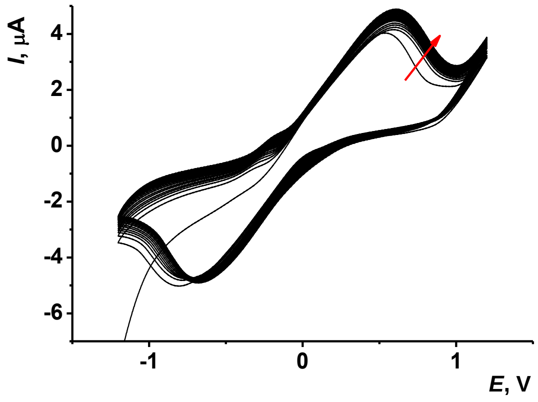

(c) (d)

Figure S2. Multiple cyclic voltammograms recorded on the carbon SCE in NADES (citric acid, glucose and water, molar ratio 1:1:6) with 0.067 M thionine. Scan rate 50 mV/s; (a) 7 cycles; (b) 15 cycles; (c) 20 cycles; and (d) 30 cycles of the potential. Arrows indicate changes with increasing number of cycles

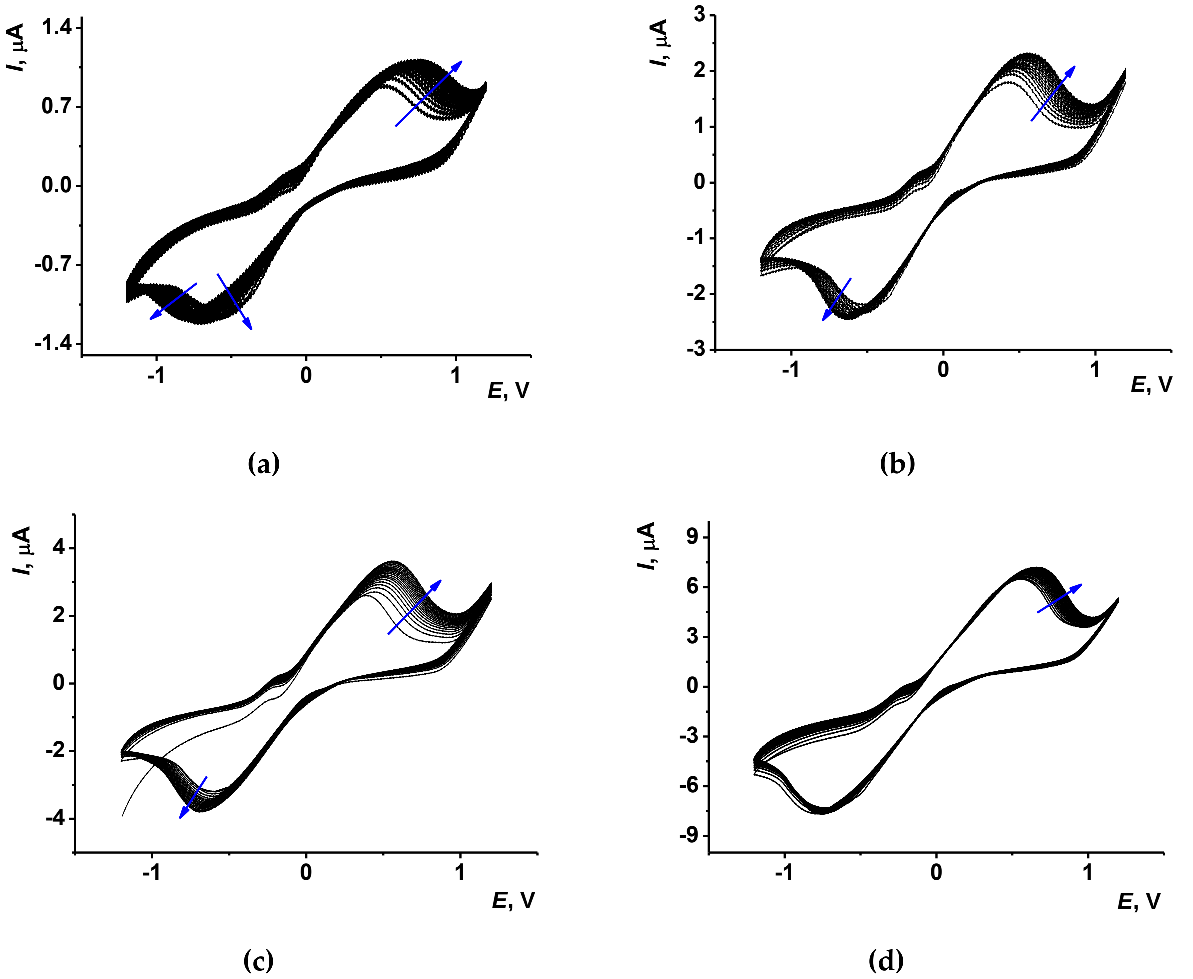

Figure S3. Multiple cyclic voltammograms recorded on the carbon SCE in NADES (citric acid, glucose and water, molar ratio 1:1:6), 20 cycles of the potential, scan rate 50 mV/s, in the presence of (a) 0.017; (b) 0.033, (c) 0.067 and (d) 0.10 M of thionine. Arrows indicate changes with increasing number of cycles.

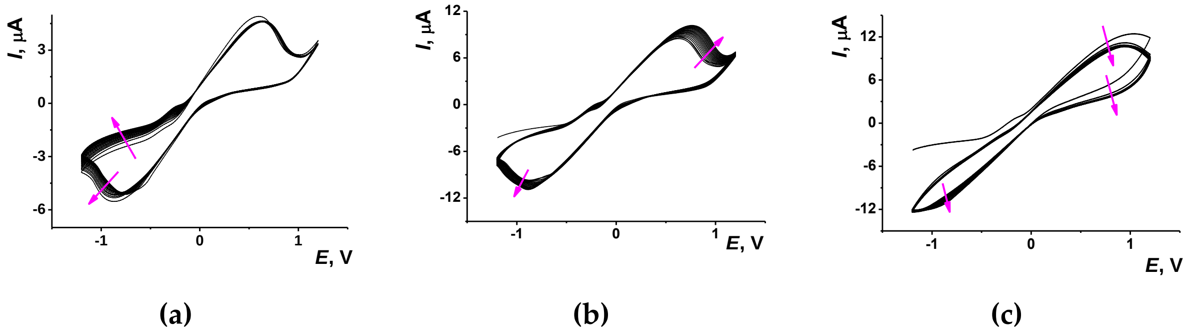

Figure S4. Multiple cyclic voltammograms recorded on the carbon SCE in NADES (citric acid, glucose and water, molar ratio 1:1:6) containing 0.1 M thionine, 20 cycles, at the scan rate (a) 50, (b) 100, and (c) 300 mV/s. Arrows indicate changes with increasing number of cycles.

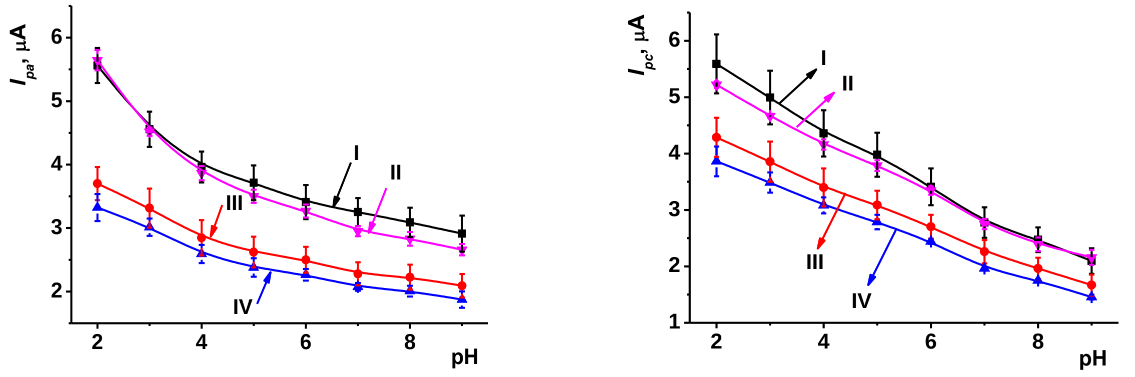

(a) (b)

Figure S5. The pH dependence of the anodic (a) and cathodic (b) peak currents of the PTN_{aq} with no signal stabilization (I), open-circuit (non-current) stabilization (II), tenfold consecutive scans in a single drop of HEPES (III), and tenfold consecutive scans in the drop of HEPES refreshed after each scan (IV). Electropolymerization of thionine at SPE in 0.1 M HEPES containing 0.03 M NaCl, pH 7.0, 100 mV/s, 20 cycles

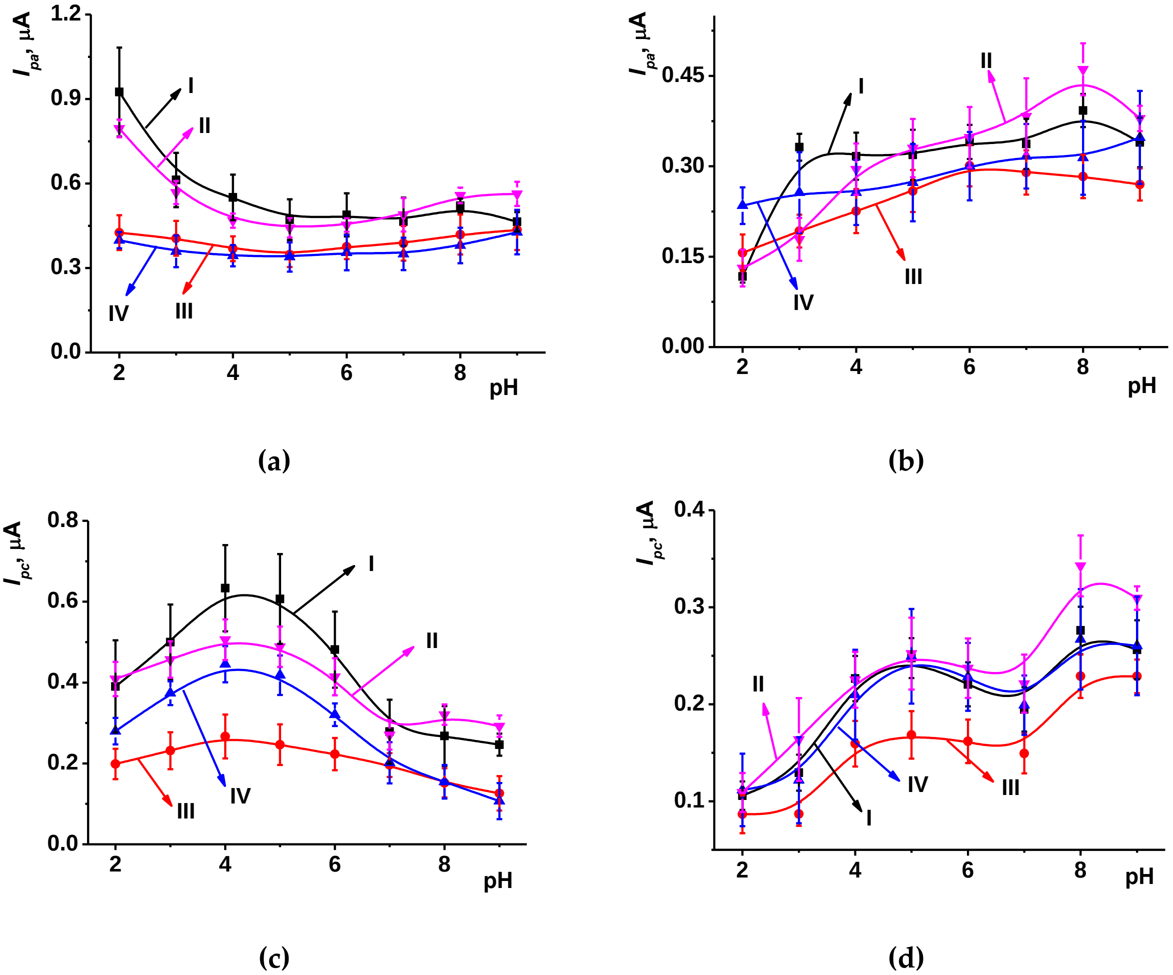

Figure S6. The pH dependence of the anodic peak current of the monomer (a), polymer (b), of the cathodic peak currents of the monomer (c), polymer (d) for the PTN_{NADES}, with no signal stabilization (I), open-circuit (non-current) stabilization (II), tenfold consecutive scans in a single drop of HEPES (III), and tenfold consecutive scans in the drop of HEPES refreshed after each scan (IV). Electropolymerization of thionine on SPE in 0.1 M HEPES containing 0.03 M NaCl, pH 7.0, 100 mV/s, 20 cycles

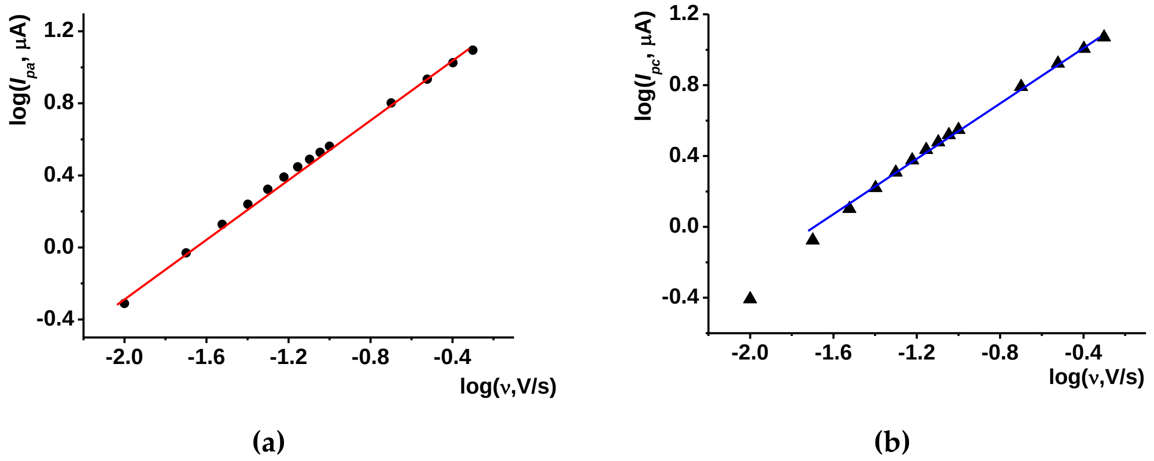

Figure S7. The scan rate (ν) dependence of the anodic I_{pa} (a) and cathodic I_{pc} (b) peak currents of the PTN_{aq} in 0.1 M HEPES containing 0.03 M NaCl, pH 7.0, scan rate 10 - 500 mV/s

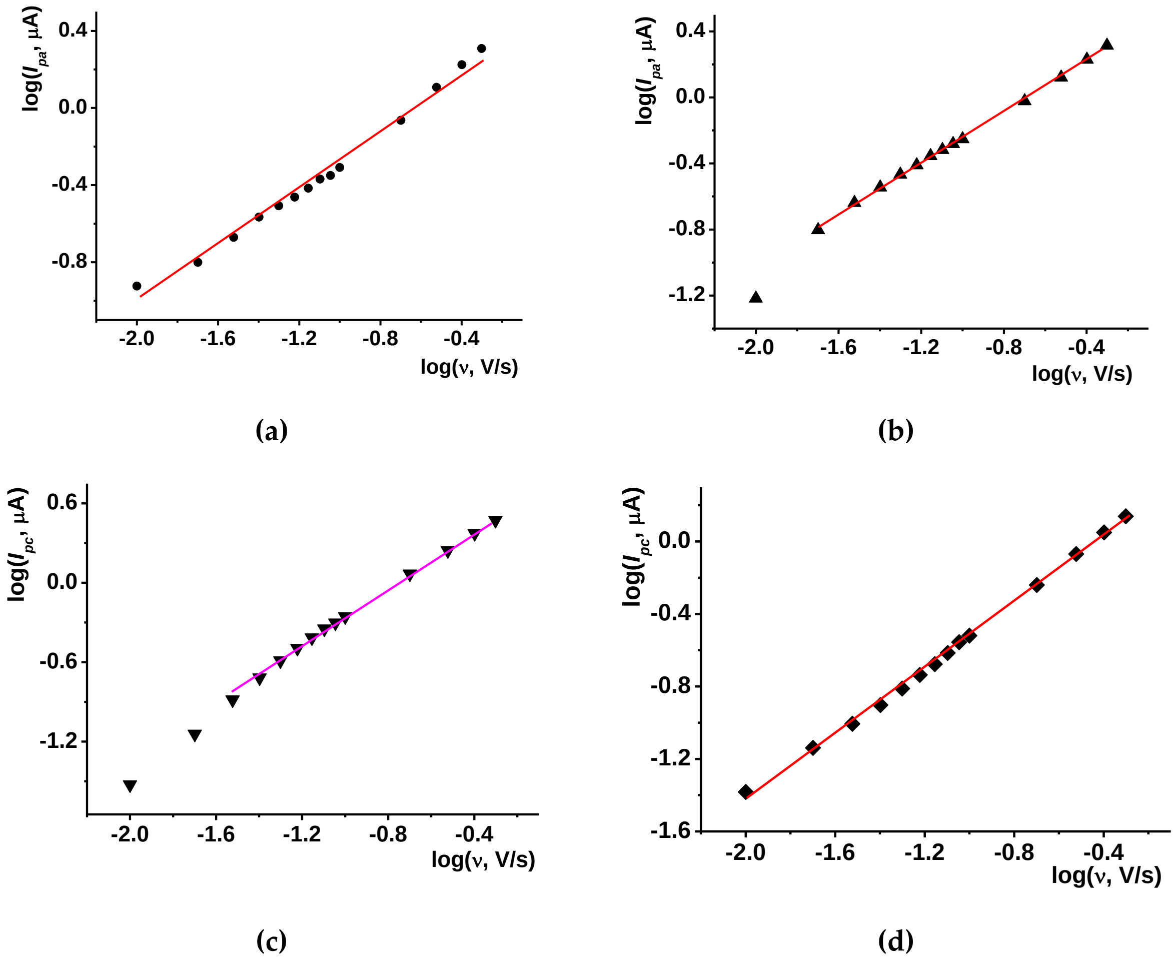

Figure S8. The scan rate dependence of the anodic (I_{pc}) peak current of the monomer (a), polymer (b), of the cathodic (I_{pc}) peak current of the monomer (c), polymer (d) for the PTN_{NADES} in 0.1 M HEPES containing 0.03 M NaCl, pH 7.0, 10 - 500 mV/s

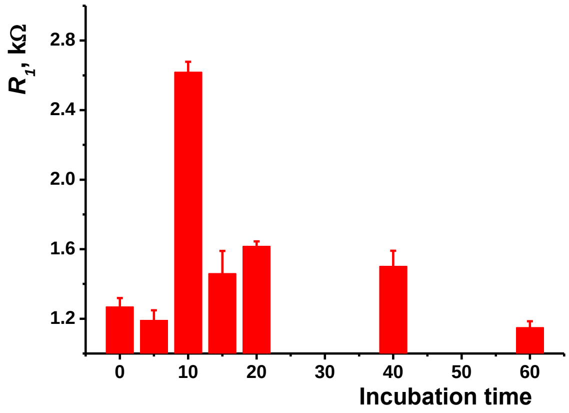

Figure S9. The charge transfer resistance on the modifying layer – solution interface for various incubation time in 10 μM epirubicin, PTNNADES in 0.1 M HEPES containing 0.03 M NaCl, pH 7.0, in presence of 0.01 M $[Fe(CN)_6]^{3-/4-}$ redox probe
